# Pathology Report Interpretation and Disease Diagnosis Using Fuzzy Logic Embedded in an Artificial Intelligence Framework: A New Paradigm for Digital Technologies

**DOI:** 10.7759/cureus.71148

**Published:** 2024-10-09

**Authors:** Nisanth K Nambison, D. P Singh, Rakesh Mehar, Smita N Nambison, Hariom Sharma, Divyansh Sharma, Eva Nambison

**Affiliations:** 1 Research, Government Homeopathic Medical College and Hospital, Bhopal, IND; 2 General Medicine, LN Medical College and Research Center, Bhopal, IND; 3 Pathology, Mahatma Gandhi Memorial (MGM) Medical College, Indore, IND; 4 Medical Technology, Neshlin Medtech Private Limited, Bhopal, IND; 5 Civil Engineering, Maulana Azad National Institute of Technology Bhopal, Bhopal, IND; 6 Biomedical Engineering, Indian Institute of Technology Varanasi, Varanasi, IND; 7 Data Science, Indian Institute of Technology Madras, Chennai, IND

**Keywords:** artificial intelligence, blood pathology, diagnostics, differential diagnosis, digital pathology, disease diagnosis, fuzzy logic, laboratory test, pathology laboratory report interpretation, technologies

## Abstract

Introduction

Accurate diagnosis is essential for effective treatment in healthcare settings. Digital technologies have revolutionized medical diagnostics, particularly in pathology laboratory report analyses, by enhancing speed, quality, and precision. Diagnostic pathology has advanced significantly with digital imaging, AI algorithms, and computer-assisted techniques. Traditional machine learning methods require extensive training data, struggle with decision-making flexibility, and lack interpretability when handling new data.

Methods

This paper introduces a novel approach to address these challenges: a fuzzy logic-based system for interpreting pathology laboratory reports and diagnosing diseases, while considering the restrictions of engineered interpretations so that the degree of reliability of the output of the system is at par with human perception.

Results

The analysis highlights the impressive accuracy of the fuzzy logic-based diagnosis system, which is closely aligned with professional diagnoses. Notably, it correctly identified "normal" with an 83% probability, showcasing its potential for early disease detection. Performance evaluations indicated that the precision, recall, and *F*-measure significantly improved as the number of probable diagnoses considered increased. Furthermore, the system exhibited enhanced predictive accuracy for disease recurrence when incorporating multiple probable diagnoses, underscoring its robust clinical effectiveness.

Conclusion

A fuzzy logic-based system within an AI framework for interpreting pathology laboratory reports and diagnosing 17 diseases across various demographics is presented. The performance of the algorithm, assessed using specialized classification metrics, showed stable and satisfactory results. The analysis revealed a direct correlation between the number of probable diagnoses considered and improved diagnostic accuracy with enhancements in precision, recall, and *F*-measure. Broadening the diagnostic scope optimizes immediate patient care and long-term treatment planning, potentially enhancing patient outcomes and healthcare quality.

## Introduction

In today's healthcare environment, diagnostic precision is crucial for the effective treatment of diseases [1]. The introduction of digital technology has revolutionized medical diagnostics, particularly the analysis of pathology laboratory reports [2,3]. This shift marks a significant advancement by providing tools that enhance the speed, quality, and accuracy of diagnoses. Diagnostic pathology has evolved remarkably by incorporating digital imaging, sophisticated artificial intelligence (AI) algorithms, and computer-aided diagnostic methods. These innovations have significantly improved the capabilities of computational histopathology and AI-driven diagnostics, establishing new benchmarks for disease identification and management [4].

Despite these advancements, traditional AI techniques, which rely heavily on machine learning algorithms, have notable limitations. They depend significantly on the quality of the training data and often lack transparency in their decision-making processes when dealing with new data samples [5,6]. This lack of clarity poses a significant issue, as it makes understanding how AI systems reach their conclusions challenging [7]. Additionally, the complex nature of pathological data, grounded in specific and experimentally validated interpretations, adds another layer of complexity [8]. Conventional AI methods, which aim to extract as much information as possible from training data, often struggle with these constraints, leading to arbitrary decisions if they do not strictly adhere to established interpretations [9,10]. Given the critical importance of medical diagnoses, ensuring that AI-driven decisions align with recognized medical standards is essential.

Therefore, a new approach is required to overcome these challenges. This study introduces an innovative solution, a fuzzy logic-based system [11], designed for interpreting pathology laboratory reports and diagnosing diseases. Unlike traditional AI models, this system operates within the boundaries of predefined interpretations, ensuring that each diagnosis is as reliable and interpretable as those made by human professionals. By adhering to established medical standards, this system reduces the risk of arbitrary decision-making in AI-driven diagnostics, representing a significant step forward in integrating AI technologies in healthcare.

This system was designed to handle the uncertainties inherent in medical data and mimic the interpretive nuances of human judgment. Considering the broader range of possible diagnoses, it is hypothesized that the system can achieve higher diagnostic precision, recall, and *F*-measure across various diseases, thus providing more accurate and comprehensive diagnostic outcomes than conventional methods.

The innovation of this study lies in the integration of fuzzy logic into the interpretation of pathology laboratory reports and disease diagnosis. Unlike traditional AI models, which depend heavily on the quality and quantity of training data and often function as opaque "black boxes," this fuzzy logic-based system respects predefined interpretations of pathology laboratory data. This ensures that the diagnostic decisions are reliable and interpretable for human judgment. By addressing uncertainties and offering transparent decision-making processes, this system addresses the inherent complexities and limitations of conventional AI techniques, thereby providing a significant advancement in computational histopathology and AI-driven diagnostics.

## Materials and methods

Fuzzy logic offers a promising alternative to address the challenges of existing diagnostic techniques [11,12]. Fuzzy logic, with its ability to handle uncertainty and provide interpretable decisions, is particularly suited for medical diagnostics, in which decisions must be made with partial or ambiguous information.

Fuzzy logic system

**Figure 1:**
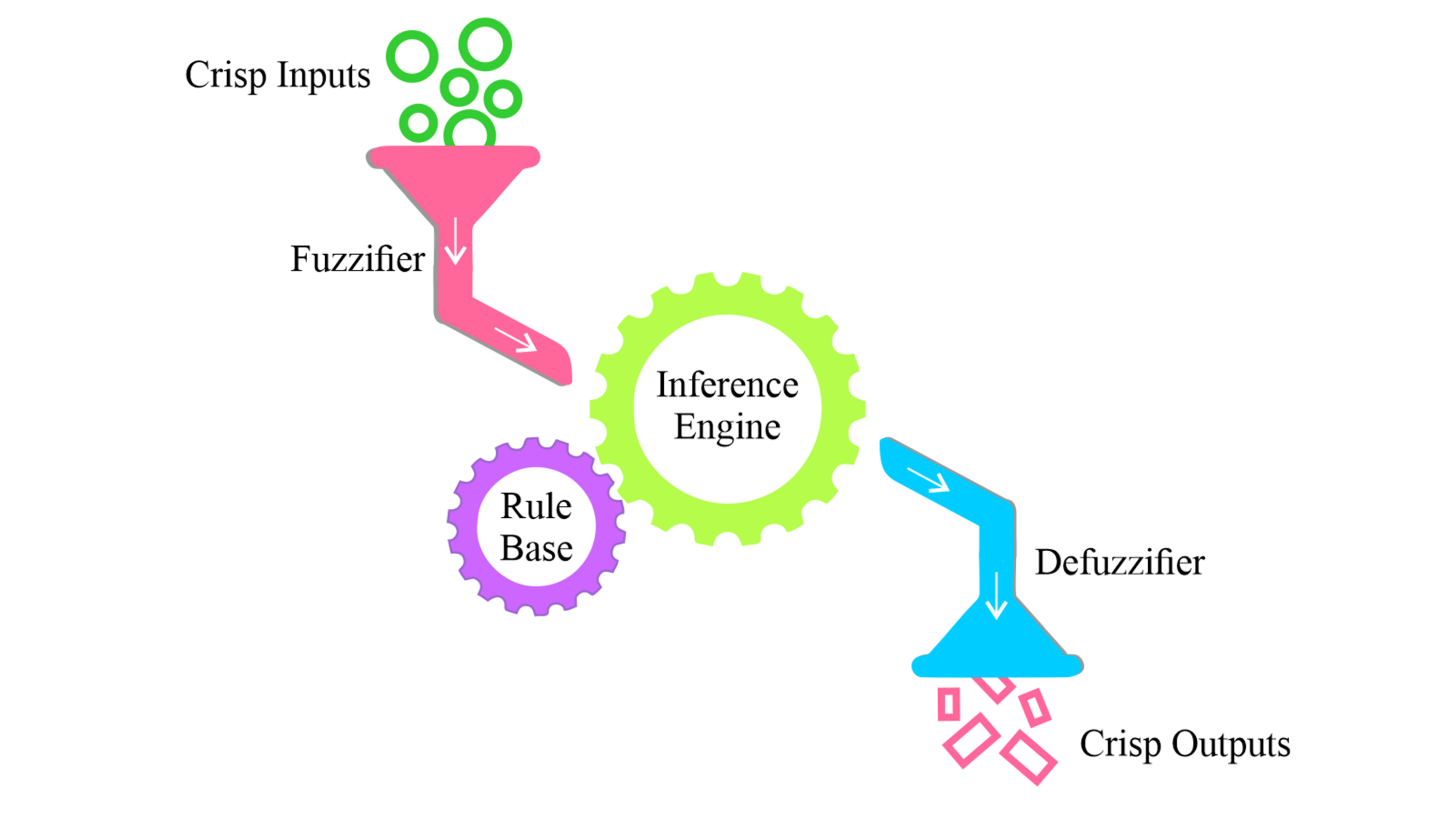
Typical architecture type-I fuzzy logic controller (FLC) system Image credit: the authors

A typical architecture of a type-I fuzzy logic system, first proposed by Professor Lofti Zadeh [13], is presented in Figure 1, where crisp inputs are fed into the system and fuzzified by the fuzzifier, and the fuzzy rule-based inference engine arrives at an inference, which is then defuzzified by the defuzzifier for crisp output.

Crisp Input

This is the input interface of the fuzzy logic controller (FLC) with the external environment from which it receives crisp data for decision-making or control.

Fuzzification

Fuzzification is a method of translating crisp values in terms of degree of membership using fuzzy sets. The membership degree is determined by the membership functions. Fuzzification enables the linguistic representation of crisp data. 

Fuzzy Rule Base

This component stores rules that linguistically connect fuzzy inputs and outputs. It typically comprises an "IF-THEN" rule structure. For example, the rule base of an automatic fan speed control system may contain the following rules: If the fan speed is "LOW," then set the speed to "HIGH.” If the speed is "MEDIUM", then the speed is set to "MEDIUM." If the speed is "HIGH,” then the speed is set to "LOW.” Here, only three rules were presented. However, a practical system may contain hundreds of rules, and defining these rules requires expert knowledge of the field of application. 

Inference Engine

Fuzzy outputs are generated by processing fuzzy inputs according to rules defined in the rule base.

Defuzzifier

The inference engine produces fuzzy outputs, which must be transformed into crisp values before they can be used with any nonfuzzy system. A defuzzifier was used to perform this conversion. Defuzzification can be carried out in many ways, such as using the center of gravity, center of area, middle of the maxima, and mean of the maxima.

Proposed approach

**Figure 2:**
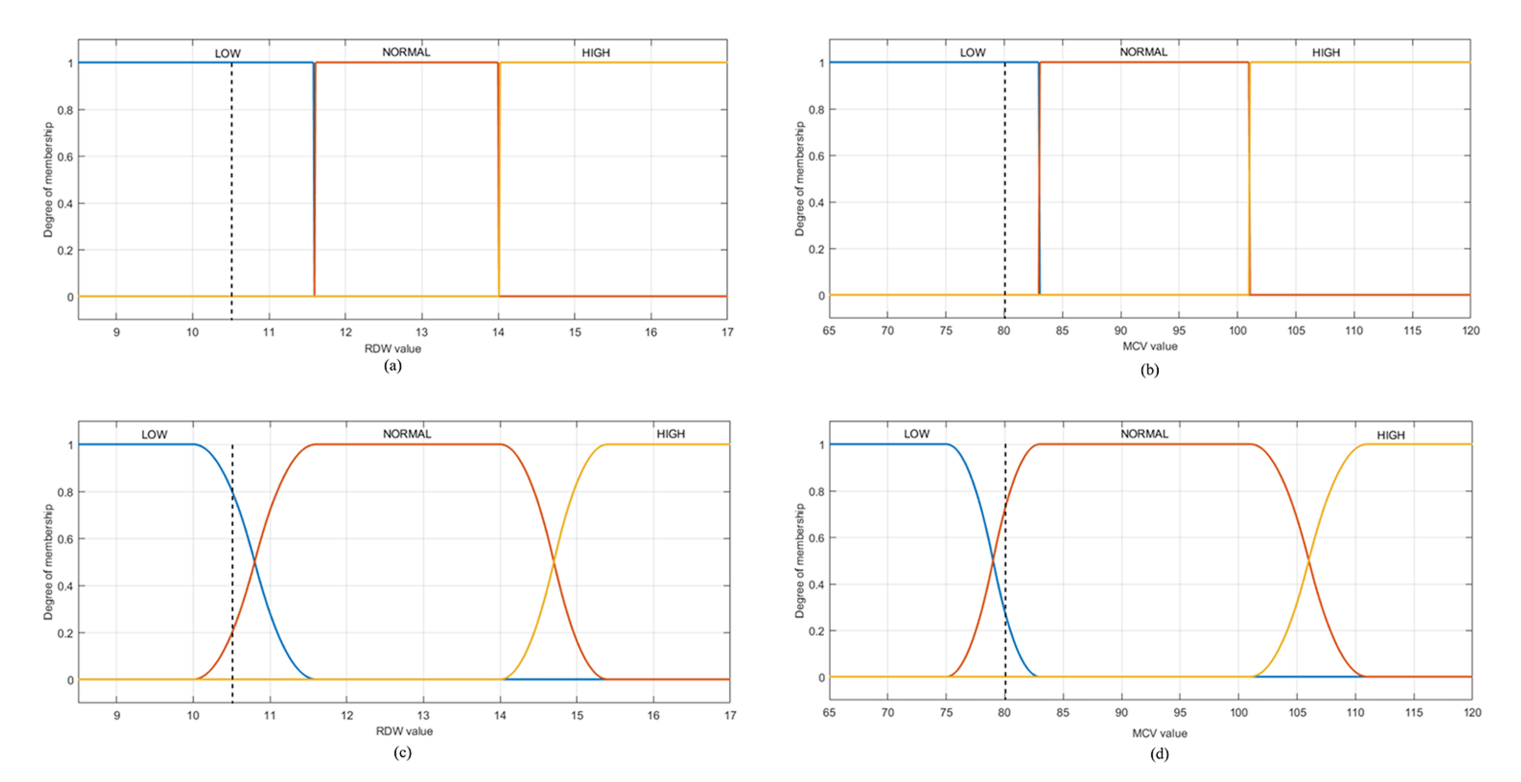
Illustrative categorization plot of RDW and MCV values using crisp set theory (a, b) and fuzzy set theory (c, d) RDW: red cell distribution width; MCV: mean corpuscular volume Image credit: the authors

The fuzzy set provides an advantage over the conventional crisp set by assigning the same variable to multiple sets or categories, based on the degree of membership calculated using the membership function. This approach converts the problem of an abrupt change in the category into a gradual transition, where the parameter may belong to multiple categories defined by the membership function in the transition range. An illustrative example of this categorization is shown in Figure 2.

As shown in Figure 2, the categorization of pathology test values 10.5 and 80.0 for red cell distribution width (RDW) and mean corpuscular volume (MCV) using the crisp set method, both RDW and MCV are categorized as low; therefore, only one possible disease can be diagnosed. On the other hand, using a fuzzy set, the RDW can be categorized as both low and normal, with membership (or possibility) of 0.8 and 0.2, respectively. Similarly, the MCV can also be categorized as low and normal, with membership (or possibility) of 0.3 and 0.7, respectively. Thus, with a fuzzy set, there are four diagnoses that differ from those with a crisp set.

Proposed evaluation model

**Figure 3:**
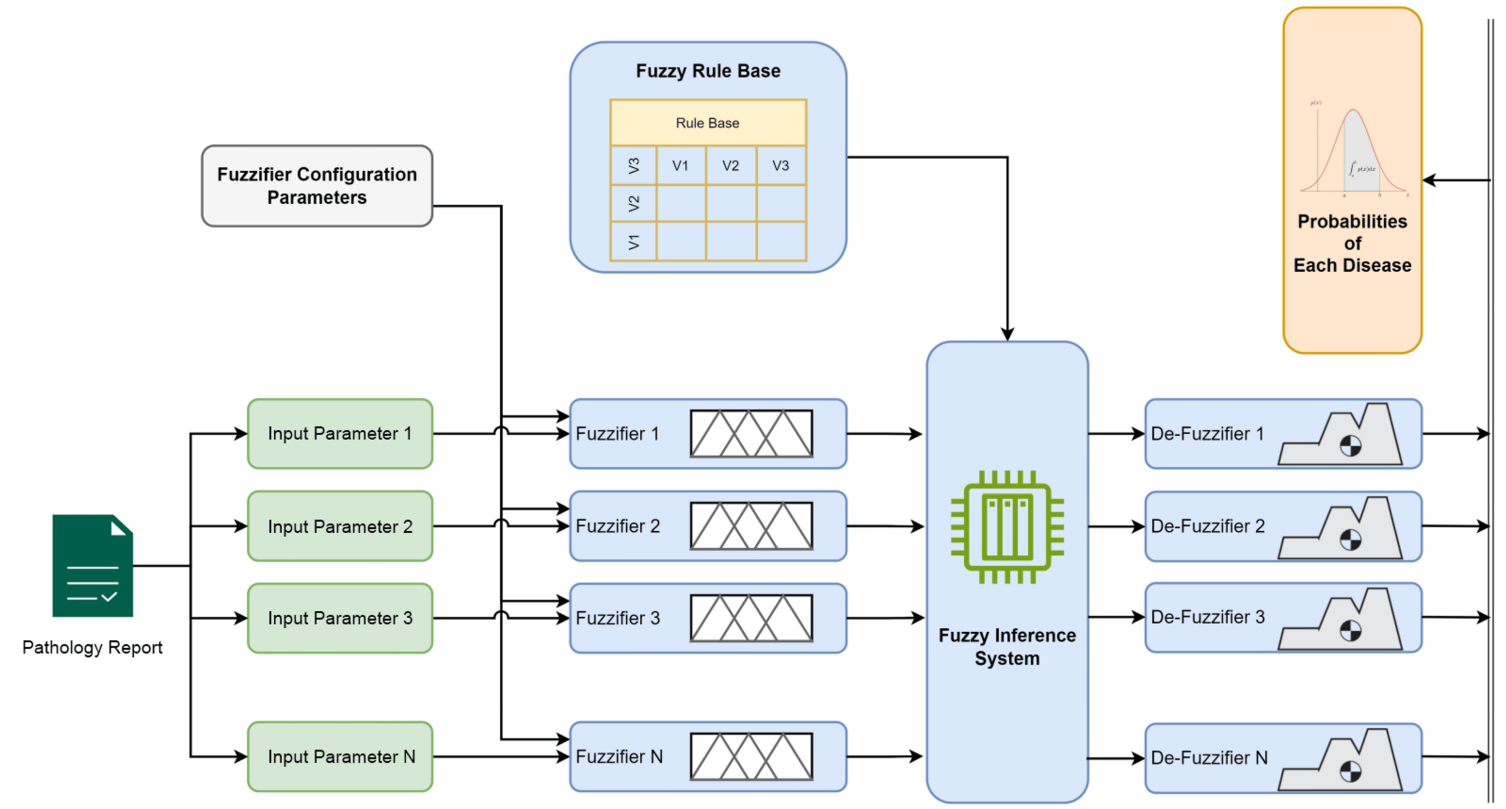
Illustrative block diagram of the proposed fuzzy-based disease diagnosis system Image credit: the authors

Based on the approach proposed in the previous section, we developed a complete evaluation model to test the proposed approach by using actual pathology laboratory data. An illustrative block diagram of the proposed fuzzy logic-based disease diagnosis system evaluation model is shown in Figure 3, which can be described as follows.

User Input

The process begins with the user input, where the specific configuration parameters for the fuzzifier are defined. These parameters include the overlapping locations of the membership functions, the number of membership functions, and the type of membership functions.

Pathology Report

The system receives input parameters derived from pathology laboratory reports. These parameters could be various metrics or biomarkers reported in pathology laboratory reports such as RDW and MCV.

Dataset Description

Table 1 presents the dataset used to evaluate the proposed technique experimentally. Four input parameters were used to diagnose the possibility of the 17 diseases. Participants were further divided into sex and age groups.

**Table 1:** Table showing dataset description

Category	Label	Details
Input parameters	MCV	Mean corpuscular volume
	RDW	Red blood cell distribution width
	Hb	Hemoglobin
	PCV	Packed cell volume
Diseases	D0	Normal
	D1	Thalassemia carrier
	D2	Anemia of chronic disease
	D3	Iron deficiency anemia
	D4	Hemoglobin H disease
	D5	Sickle cell β thalassemia
	D6	Myelodysplastic syndrome
	D7	Aplastic anemia
	D8	Megaloblastic anemia
	D9	Immune hemolytic anemia
	D10	Sickle cell trait
	D11	Hereditary spherocytosis
	D12	Sideroblastic anemia
	D13	Myelofibrosis
	D14	Sickle cell anemia
	D15	Polycythemia vera
	D16	Dehydration
	D17	Anemia
Gender groups	G1	Male
	G2	Female
	G3	Female (pregnant)
Age groups	A1	1 day to 2 months
	A2	2 months to 6 months
	A3	6 months to 6 years
	A4	6 years to 18 years
	A5	Over 18 years

Input Parameters (1 to N)

Each parameter from the pathology report was used as the input. The diagram shows multiple input parameters, labeled from to, indicating that the system can handle numerous inputs.

Fuzzifiers (1 to N)

Each input parameter was processed using a fuzzifier. The fuzzifier converts crisp input values into fuzzy values based on the membership functions and configuration parameter set.

Fuzzy Rule Base

The fuzzy values are then used in conjunction with a fuzzy rule base. The rule base contains a set of rules that describe how to infer disease probabilities from the fuzzy input values. These rules are formulated using expert knowledge and empirical data.

Fuzzy Inference System

The core of the diagnosis system is the fuzzy inference system (FIS). The FIS applies fuzzy rules to fuzzified inputs to derive the fuzzy outputs. It processes the information and infers the degree to which each disease is present based on the input parameters.

Defuzzifiers (1 to N)

The fuzzy outputs from the inference system are then converted back into crisp values by the defuzzifiers. Each defuzzifier corresponds to an input parameter and produces a specific output related to the likelihood of disease.

Probabilities of Each Disease

Finally, defuzzified values represent the probabilities of each disease. Based on the pathology report and fuzzy logic interpretation, these probabilities indicated the likelihood of each disease being present.

Performance evaluation metrics

The utilization of performance indicators is advantageous when comparing diverse classification models and machine learning methodologies. Numerous evaluation metrics were used to assess the capabilities of the classifiers. The majority of these metrics are derived from the confusion matrix as they encompass all pertinent information regarding the algorithm and classification rule performance.

**Figure 4:**
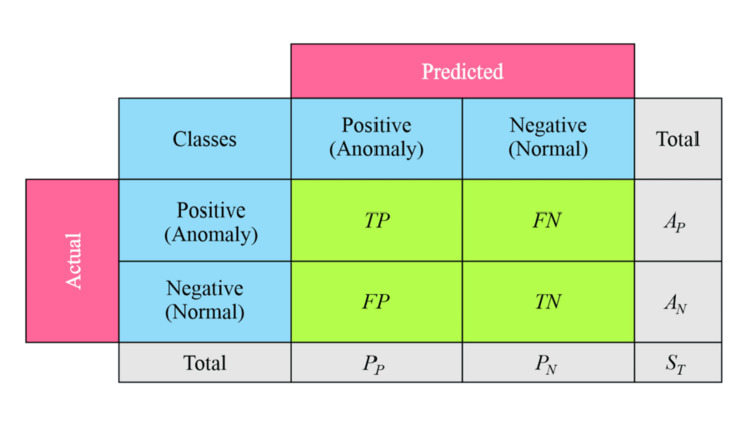
Two-class confusion matrix TP: true positive; TN: true negative; FP: false positive; FN: false negative; *A*_P_: actual positive instances; *A*_N_: actual negative instances; *P*_P_: predicted positive instances; *P*_N_: predicted negative instances; *S*_T_: total number of instances Image credit: the authors

In Figure 4, a two-class confusion matrix is shown, where true positive (TP), true negative (TN), false positive (FP), and false negative (FN) represent the true positive, true negative, false positive, and false negative instances, respectively; *A*_P_, *A*_N_, *P*_P_, and *P*_N_ represent the numbers of actual positive instances, actual negative instances, predicted positive instances, and predicted negative instances, respectively; and *S*_T_ represents the total number of instances in the test dataset.

Based on these terms, the four common measures are as follows:

$Accuracy = \frac{(TP+TN)}{(TP+TN+FP+FN)}$ (1)

$Precision = \frac{TP}{(TP+FP)}$ (2)

$Recall = \frac{TP}{(TP+FN)}$ (3)

$F = 2∙\frac{Precision∙Recall}{(Precision+Recall)}$ (4)

These measurements can be extended to multiclass scenarios [14,15].

This study did not involve human participants or tissue samples. Instead, we used anonymized laboratory values obtained from the pathology laboratories and our own clinical practice. According to the "Guidance on ethical requirements for laboratory validation testing by the Bioethics Unit of the Indian Council of Medical Research," studies that rely on anonymized data present no risk of harm or discomfort to patients. As the values were anonymized, the privacy and confidentiality of the individuals were fully protected. There was no requirement for informed consent and formal ethics approval [16].

## Results

The evaluation of the utilization of fuzzy logic within an artificial intelligence framework for disease diagnosis through the interpretation of pathology laboratory reports elucidated a spectrum of potential applications. Herein, we present the findings of the current study.

Diagnosis results

Table 2 presents the distribution of samples for various diseases categorized by sex and specific subgroups within the male and female populations. Diseases were labeled from D0 to D17. Each disease was further broken down by the number of samples in five age groups (A1 to A5) for both males and females, including a special subcategory "Female (P)" for pregnant females.

**Table 2:** Number of samples for each disease in the test dataset D represents disease, which is labeled from D0 to D17. A represents the disease; five age groups are labeled from A1 to A5, and Female (P) represents pregnant females

Disease	Male					Female					Female (P)	Total samples
	A1	A2	A3	A4	A5	A1	A2	A3	A4	A5		
D0	2	4	5	4	12	2	2	3	3	10	4	51
D1	0	1	1	2	5	0	1	2	1	4	3	20
D2	0	0	0	2	10	0	0	0	4	5	2	23
D3	0	0	2	2	6	0	0	1	1	4	2	18
D4	0	0	0	0	1	0	0	0	0	1	0	2
D5	0	1	1	3	13	0	0	1	2	6	2	29
D6	0	0	0	0	1	0	0	0	0	1	0	2
D7	1	2	2	4	6	1	1	1	2	10	1	31
D8	0	0	1	1	2	0	0	1	1	3	7	16
D9	0	0	0	0	2	0	0	0	0	0	0	2
D10	0	1	2	2	3	0	1	1	1	2	1	14
D11	0	0	1	0	1	0	0	1	1	1	0	5
D12	0	0	0	1	1	0	0	1	1	1	0	5
D13	0	0	1	0	1	0	0	0	1	1	0	4
D14	1	1	2	3	5	0	1	2	2	3	2	22
D15	0	0	1	1	2	0	0	0	1	1	1	7
D16	1	2	2	6	6	2	1	3	3	7	4	37
D17	0	0	0	4	4	0	0	0	3	4	8	23

Table 3 provides a comprehensive comparison of the proposed fuzzy logic-based diagnosis system predictions and professional diagnoses. This table includes the eight most probable diseases identified by the system, with their corresponding probabilities compared to the diseases diagnosed by professionals, the results from further diagnostic procedures, and any diseases that appeared later in the patient's medical records.

**Table 3:** Disease diagnosis comparison table for eight most probable diseases diagnosed by the system MCV: mean corpuscular volume; RDW: red blood cell distribution width; Hb: hemoglobin; PCV: packed cell volume; G1: male; G2: female; G3: female (pregnant). Age groups A1: 1 day to 2 months; A2: 2 months to 6 months; A3: 6 months to 6 years; A4: 6 years to 18 years; A5: over 18 years

Gender	Age	MCV (fL)	RDW (fL)	Hb (g/dL)	PCV (%)	Eight most probable diseases diagnosed by system								Two most probable diseases diagnosed by professional		Results of further diagnosis	Disease appears later
G2	A5	89.6	13.6	12.0	39.5	D0 (0.83)	--	--	--	--	--	--	--	D0	D0	D0	D0
G1	A5	83.3	14.4	12.1	40.5	D0 (0.60)	D17 (0.33)	--	--	--	--	--	--	D0	D0	D0	D0
G2	A5	78.6	17.7	9.1	35.2	D17 (0.69)	D3 (0.68)	D4 (0.68)	D5 (0.68)	D8 (0.32)	D12 (0.32)	D13 (0.32)	D14 (0.32)	D17	D3-5	D3	D3
G2	A5	90.0	13.8	12.9	39.7	D0 (0.84)	--	--	--	--	--	--	--	D0	D0	D0	D0
G1	A5	84.3	13.8	12.6	40.8	D0 (0.80)	D17 (0.20)	--	--	--	--	--	--	D0	D0	D17	D17
G2	A5	83.4	14.9	13.2	41.1	D0 (0.50)	--	--	--	--	--	--	--	D0	D0	D0	D0
G1	A5	84.4	14.0	14.5	42.9	D0 (0.84)	--	--	--	--	--	--	--	D0	D0	D0	D0
G1	A5	115.8	17.6	8.4	26.4	D8 (0.84)	D9 (0.84)	D17 (0.50)	--	--	--	--	--	D17	D8-9	D9	D9
G1	A5	100.7	14.4	15.2	44.5	D0 (0.60)	--	--	--	--	--	--	--	D0	D0	D0	D0
G2	A5	85.8	13.5	11.6	33.3	D1 (0.41)	D10 (0.41)	D11 (0.41)	D17 (0.38)	--	--	--	--	D0	D17	D0	D0
G2	A5	70.0	18.3	9.5	31.8	D3 (0.84)	D4 (0.84)	D5 (0.84)	D17 (0.84)	--	--	--	--	D3	D17	D5	D5
G2	A5	87.7	15.5	10.8	34.1	D8 (0.84)	D12 (0.84)	D13 (0.84)	D14 (0.84)	D17 (0.50)	--	--	--	D8	D12	D14	D14
G2	A5	70.3	18.6	8.9	31.7	D3 (0.84)	D4 (0.84)	D5 (0.84)	D17 (0.84)	--	--	--	--	D3	D4-5	D5	D5
G1	A5	86.3	11.9	9.6	25.9	D1 (0.84)	D10 (0.84)	D11 (0.84)	D17 (0.50)	--	--	--	--	D1	D11	D10	D10
G2	A5	69.7	17.4	9.8	31.3	D3 (0.84)	D4 (0.84)	D5 (0.84)	D17 (0.84)	--	--	--	--	D3	D4-5	D5	D5
G1	A5	73.4	14.5	11.6	36.1	D1 (0.48)	D2 (0.48)	D3 (0.48)	D4 (0.48)	D5 (0.48)	D17 (0.48)	--	--	D17	D3	D17	D17
G1	A5	88.0	13.0	7.4	20.5	D1 (0.84)	D10 (0.84)	D11 (0.84)	D17 (0.50)	--	--	--	--	D1	D17	D11	D11
G2	A5	78.4	14.9	11.4	33.0	D3 (0.59)	D4 (0.59)	D5 (0.59)	D17 (0.59)	D8 (0.26)	D12 (0.26)	D13 (0.26)	D14 (0.26)	D3	D17	D5	D5
G2	A5	59.9	18.2	11.6	37.5	D17 (0.39)	D3 (0.30)	D4 (0.30)	D5 (0.30)	--	--	--	--	D0	D17	D17	D17
G1	A5	102.5	14.0	8.9	28.8	D6 (0.84)	D7 (0.84)	D17 (0.50)	--	--	--	--	--	D7	D17	D6	D6
G2	A5	75.3	14.0	10.6	32.9	D1 (0.84)	D2 (0.84)	D17 (0.84)	--	--	--	--	--	D1	D17	D2	D2
G1	A5	93.0	13.0	18.0	52.0	D17 (0.50)	--	--	--	--	--	--	--	D0	D17	D17	D17

For instance, the first entry of a five-year-old patient with pathology values (MCV, 89.6; RDW, 13.6; Hb, 12.0; PCV, 39.5) showed that the system predicted the disease D0 (normal) with a probability of 0.83, which matched the professional diagnosis. The system consistently identified D0 as the most probable disease across multiple patients with different pathological profiles, indicating a strong alignment with professional diagnoses in normal cases.

Another notable case involved a patient with an MCV of 78.6, RDW of 17.7, Hb of 9.1, and PCV of 35.2, where the system predicted D17 (dehydration) with a probability of 0.69 and D3 (iron deficiency anemia) with a probability of 0.68. Professional diagnosis confirmed D17 and indicated further testing for D3, showcasing the system's ability to accurately identify both potential primary and secondary diseases.

The table also highlights instances in which the system's predictions preemptively identified diseases that appeared in later stages of diagnosis, such as thalassemia carriers and sickle cell anemia, indicating its potential for early detection and long-term prognostic capabilities. Overall, Table 2 demonstrates the fuzzy logic-based system's high accuracy and reliability in alignment with professional diagnoses, robustness in handling multiple probable diseases, and capability for early disease detection. This table underscores the system's effectiveness and potential applications in clinical settings, providing significant advantages in terms of diagnostic precision and early intervention.

Table 4 provides a detailed performance evaluation of the proposed fuzzy logic-based diagnosis system, focusing on the precision, recall, and *F*-measure for each disease when considering different numbers of probable diagnoses (*N*). The diseases assessed included common conditions such as normal (D0), thalassemia carrier (D1), and anemia of chronic disease (D2).

**Table 4:** Performance evaluation results for precision, recall, and F-measures for each disease considering N most probable diagnosis

Disease	Precision considering *N* most probable diseases diagnosed by system			Recall considering *N* most probable diseases diagnosed by system			*F*-measure considering *N* most probable diagnosis diseases diagnosed by system		
	*N* = 1	*N* = 3	*N* = 5	*N* = 1	*N* = 3	*N* = 5	*N* = 1	*N* = 3	*N* = 5
Normal	0.786	0.889	1.000	0.647	0.784	0.922	0.710	0.833	0.959
Thalassemia carrier	0.538	0.727	0.857	0.700	0.800	0.900	0.609	0.762	0.878
Anemia of chronic disease	0.652	0.692	1.000	0.652	0.783	0.957	0.652	0.735	0.978
Iron deficiency anemia	0.611	0.667	1.000	0.611	0.778	0.944	0.611	0.718	0.971
Hemoglobin H disease	0.720	0.767	0.900	0.621	0.793	0.931	0.667	0.780	0.915
Sickle cell β thalassemia	0.250	0.556	1.000	0.667	0.833	1.000	0.364	0.667	1.000
Myelodysplastic syndrome	0.615	0.818	0.786	0.667	0.750	0.917	0.640	0.783	0.846
Aplastic anemia	0.760	0.926	0.931	0.613	0.806	0.871	0.679	0.862	0.900
Megaloblastic anemia	0.588	0.857	0.875	0.625	0.750	0.875	0.606	0.800	0.875
Immune hemolytic anemia	0.667	0.750	0.929	0.643	0.750	0.929	0.655	0.750	0.929
Sickle cell trait	0.529	0.579	1.000	0.643	0.786	0.929	0.581	0.667	0.963
Hereditary spherocytosis	0.636	0.600	0.714	0.636	0.818	0.909	0.636	0.692	0.800
Sideroblastic anemia	0.556	0.600	0.778	0.625	0.750	0.875	0.588	0.667	0.824
Myelofibrosis	0.733	0.650	0.833	0.647	0.765	0.882	0.688	0.703	0.857
Sickle cell anemia	0.714	0.850	0.909	0.682	0.773	0.909	0.698	0.810	0.909
Polycythemia vera	0.583	0.789	0.810	0.737	0.789	0.895	0.651	0.789	0.850
Dehydration	0.676	1.000	0.943	0.622	0.757	0.892	0.648	0.862	0.917
Anemia	0.682	0.857	0.913	0.652	0.783	0.913	0.667	0.818	0.913

For each disease, the table presents the metrics for three scenarios: *N *= 1, *N *= 3, and *N *= 5, indicating the performance of the system when considering the top one, three, and five probable diseases. The precision for D0 (normal) improved from 0.786 at *N *= 1 to a perfect 1.000 at *N *= 5, indicating increased accuracy of the system with more comprehensive diagnostic considerations. Similarly, the recall for D0 increased from 0.647 to 0.922 and the *F*-measure from 0.710 to 0.959, reflecting a significant enhancement in the system’s diagnostic capability.

Other diseases showing similar trends have been observed for other diseases. For example, thalassemia carrier (D1) showed an increase in precision from 0.538 at *N* = 1 to 0.857 at *N* = 5, with corresponding improvements in recall (from 0.700 to 0.900) and *F*-measure (from 0.609 to 0.878). Anemia of chronic disease (D2) exhibited an increase in precision from 0.652 at *N* = 1 to a perfect 1.000 at *N* = 5, recall from 0.652 to 0.957, and *F*-measure from 0.652 to 0.978.

Table 5 presents the results of predicting the probability of diagnosed diseases that appeared during further examinations. This table evaluates the predictive performance of the fuzzy logic-based diagnosis system by comparing the likelihood of diseases reappearing in subsequent checks when considering the top one, three, and five probable diagnoses. For each disease, the table shows two sets of probabilities: the probability of the diagnosed disease appearing in further examination and the probability of the diagnosed disease in the near future. These probabilities were calculated for different numbers of the most probable diseases diagnosed by the system (*N* = 1, *N* = 3, and *N* = 5). For instance, the disease "Normal" has a probability of 57.41% to reappear in further examinations when considering only the most probable diagnosis (*N* = 1). This probability increased to 62.96% for *N* = 3 and 72.00% for *N* = 5, indicating that the predictive accuracy of the system improved when more probable diagnoses were included. Similarly, the near-future probability for "Normal" starts at 62.13% for *N *= 1 and rises to 72.82% for *N* = 5. Other diseases exhibited similar trends.

**Table 5:** Results for predicting probability of diagnosed disease to appear in further examination considering N most probable diagnosis

Disease	Probability of diagnosed disease to appear in further examination, considering *N* most probable diseases diagnosed by system			Probability of diagnosed disease in near future, considering *N* most probable diseases diagnosed by system		
	*N* = 1	*N* = 3	*N* = 5	*N* = 1	*N* = 3	*N* = 5
Normal	57.41%	62.96%	72.00%	62.13%	68.55%	72.82%
Thalassemia carrier	54.17%	65.31%	67.27%	52.62%	64.32%	74.68%
Anemia of chronic disease	71.79%	79.57%	82.22%	56.48%	64.67%	74.53%
Iron deficiency anemia	50.00%	55.17%	77.08%	58.17%	64.40%	74.45%
Hemoglobin H disease	58.33%	64.42%	69.81%	56.73%	64.29%	74.72%
Sickle cell β thalassemia	50.00%	64.00%	72.55%	54.28%	64.87%	74.67%
Myelodysplastic syndrome	55.10%	66.10%	75.22%	54.41%	66.76%	72.13%
Aplastic anemia	58.02%	67.39%	82.61%	58.23%	62.34%	76.92%
Megaloblastic anemia	52.94%	60.00%	77.08%	54.13%	66.84%	74.15%
Immune hemolytic anemia	53.70%	63.27%	71.15%	58.15%	62.52%	74.24%
Sickle cell trait	54.72%	70.21%	73.59%	58.33%	66.12%	72.63%
Hereditary spherocytosis	52.94%	69.57%	77.08%	54.26%	64.45%	74.14%
Sideroblastic anemia	56.25%	60.78%	72.55%	54.21%	62.91%	74.43%
Myelofibrosis	56.25%	59.94%	77.08%	54.65%	64.29%	74.75%
Sickle cell anemia	54.17%	60.78%	73.47%	52.81%	62.16%	72.42%
Polycythemia vera	49.06%	67.57%	70.59%	52.20%	64.65%	72.33%
Dehydration	58.70%	61.11%	68.52%	54.72%	64.44%	74.72%
Anemia	58.33%	64.39%	73.08%	56.48%	62.37%	76.63%

## Discussion

Evolution of diagnostic pathology with digital technologies

The integration of digital technologies has significantly influenced diagnostic pathology. Digital imaging transforms the capture, storage, and analysis of pathological data, allowing pathologists to convert glass slides into high-resolution digital slides that can be shared electronically. This change enhances diagnostic precision and quality and facilitates remote consultations, enabling access to expert pathology services globally [17]. AI and machine learning algorithms have advanced diagnostic workflows. AI rapidly analyzes vast datasets and identifies patterns that may be missed by the human eye, which is critical for interpreting complex tissue structures and cellular patterns [18]. Machine learning models, especially deep learning algorithms, have shown remarkable success in detecting tumors, classifying cancer subtypes, and predicting disease prognosis, sometimes achieving accuracy comparable to or surpassing that of experienced pathologists [19]. However, the integration of AI with pathology presents several challenges. Large annotated datasets are required to train machine learning models, which are labor-intensive and time-consuming to compile. The "black box" nature of many AI algorithms also poses issues [10], as it is essential for clinicians to understand how AI reaches its conclusions to make informed decisions about patient care. Despite these challenges, AI continues to reduce diagnostic errors and variability and improve the overall efficiency of pathology services. Computational pathology, which combines digital imaging and quantitative image analysis, is another critical area of development. This approach extracts quantitative features from digital images to build predictive models for disease diagnosis and prognosis, thereby providing more objective and reproducible analyses than the traditional methods. The integration of digital pathology with electronic health records (EHRs) and other clinical systems has enhanced the correlation between pathological findings and clinical data, enabling more precise and personalized diagnoses and paving the way for precision medicine. Standardization and interoperability efforts by organizations such as the Digital Pathology Association and FDA are crucial for the widespread adoption of digital pathology, ensuring consistent sharing and interpretation of digital slides across different platforms and institutions.

Challenges with traditional AI in pathology

Despite these advancements, several challenges hinder AI's full potential in pathology. Large annotated datasets are essential but difficult to obtain because of the labor-intensive nature of annotating histopathological images. This scarcity limits the performance and generalizability of AI models. The "black box" nature of AI algorithms also poses a problem, as clinicians need to understand the rationale behind AI-generated diagnoses to trust and integrate them into clinical practice.

Variations in the data acquisition and preprocessing techniques can affect the performance of AI models. Pathology slides prepared using different staining protocols and scanned at various resolutions introduce inconsistencies that degrade the performance of the AI models. Standardizing these methods is essential for reliable AI performance in the clinical setting.

Another challenge is class imbalance. Many pathological conditions are rare, resulting in datasets with underrepresented classes [20]. Addressing class imbalance requires techniques, such as data augmentation and resampling, to ensure that AI models are effective across all disease classes. Regulatory and ethical concerns such as patient safety and data privacy must also be addressed to ensure that AI-based diagnostic tools comply with stringent standards.

Fuzzy logic as a solution

Fuzzy logic offers a promising alternative for addressing these challenges [11]. It handles uncertainty and provides interpretable decisions, making it well-suited for medical diagnostics. Studies have demonstrated its potential in medical diagnosis, showing its ability to incorporate imprecise and qualitative information [21].

Integrating Fuzzy Logic With AI for Pathology

Integrating fuzzy logic with AI creates a hybrid model that leverages the strengths of both technologies. This combination enhances the accuracy and reliability of the diagnostic systems and provides more accurate, efficient, and transparent diagnostic systems [22]. By addressing the challenges of traditional AI methods and offering solutions through fuzzy logic, this hybrid approach marks a significant advancement in pathological diagnostics.

Limitations

The fuzzy logic-based system for interpreting pathology laboratory reports shows great potential; however, there are a few subtle limitations. Similarly, the system's reliance on predefined fuzzy rules and membership functions may not fully capture the complexity of rare or atypical medical conditions. Setting up and fine-tuning fuzzy logic rules requires some initial time and expertise, which can present minor challenges for new clinical teams.

## Conclusions

We report, interpret, and diagnose the diseases using fuzzy logic. We tested the performance of the proposed algorithm for 17 different disease diagnoses, based on four observed pathological parameters, across subjects of different genders and age groups. Finally, we thoroughly evaluated the performance by using specialized metrics designed for classification. The experimental results reveal that the proposed algorithm performs satisfactorily and provides stable performance throughout the evaluation. Furthermore, our analysis revealed a direct correlation between the number of probable diagnoses and the diagnostic accuracy of the medical system. The marked improvement in diagnostic precision, recall, and *F*-measure, along with the predictive probabilities for future disease diagnosis, advocates a more inclusive diagnostic methodology. Adopting a broader diagnostic perspective ensures comprehensive evaluation and enhances the accuracy and reliability of medical diagnoses.

These findings strongly suggest that expanding the scope of probable diagnoses can significantly improve patient outcomes by facilitating a more accurate and holistic disease identification and prediction. This comprehensive approach to diagnosis not only optimizes immediate patient care but also contributes to more informed and effective long-term treatment planning, thereby reinforcing the critical role of extensive diagnostic considerations in enhancing the quality of healthcare delivery.
